# 2,3-Dichloro-1,4-hydro­quinone 2,3-dichloro-1,4-benzoquinone monohydrate: a quinhydrone-type 1:1 donor-acceptor [*D*—*A*] charge-transfer complex

**DOI:** 10.1107/S1600536811041377

**Published:** 2011-10-12

**Authors:** Xavier Guégano, Jürg Hauser, Shi-Xia Liu, Silvio Decurtins

**Affiliations:** a Departement für Chemie und Biochemie, Universität Bern, Freiestrasse 3, CH-3012 Bern, Switzerland

## Abstract

In the crystal structure of the title compound (systematic name: 2,3-dichloro­benzene-1,4-diol 2,3-dichloro­cyclo­hexa-2,5-diene-1,4-dione monohydrate), C_6_H_4_Cl_2_O_2_·C_6_H_2_Cl_2_O_2_·H_2_O, the 2,3-dichloro-1,4-hydro­quinone donor (*D*) and the 2,3-dichloro-1,4-benzoquinone acceptor (*A*) mol­ecules form alternating stacks along [100]. Their mol­ecular planes [maximum deviations for non-H atoms: 0.0133 (14) (*D*) and 0.0763 (14) Å (*A*)] are inclined to one another by 1.45 (3)° and are thus almost parallel. There are π–π inter­actions involving the *D* and *A* mol­ecules, with centroid–centroid distances of 3.5043 (9) and 3.9548 (9) Å. Inter­molecular O—H⋯O hydrogen bonds involving the water mol­ecule and the hy­droxy and ketone groups lead to the formation of two-dimensional networks lying parallel to (001). These networks are linked by C—H⋯O inter­actions, forming a three-dimensional structure.

## Related literature

For prototypical examples of similar organic redox systems, see: Yi *et al.* (2009*a* ▶,*b* ▶). For details concerning quinhydrone, a 1:1 hydro­quinone-quinone adduct, and a well known mol­ecular charge-transfer (CT) complex, see: Foster (1969 ▶). For structural studies of different polymorphs of quinhydrone, see: Matsuda *et al.* (1958 ▶); Sakurai (1965 ▶,1968 ▶). For details concerning quinhydrone analogues, see: Bouvet *et al.* (2006 ▶,2007 ▶); Patil *et al.* (1984 ▶); Yamamura *et al.* (2007 ▶). For a detailed computational study on the stacking energies and the electron density topology in quinhydrone, see: Gonzalez Moa *et al.* (2007 ▶). 
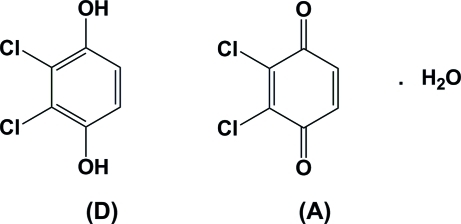


## Experimental

### 

#### Crystal data

C_6_H_4_Cl_2_O_2_·C_6_H_2_Cl_2_O_2_·H_2_O
*M* *_r_* = 373.98
Monoclinic, 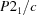

*a* = 7.15329 (14) Å
*b* = 7.19541 (15) Å
*c* = 27.2811 (5) Å
β = 92.9738 (18)°
*V* = 1402.29 (5) Å^3^
*Z* = 4
Mo *K*α radiation
μ = 0.86 mm^−1^
*T* = 173 K
0.3 × 0.2 × 0.07 mm

#### Data collection

Siemens SMART 1K CCD area-detector diffractometer
Absorption correction: multi-scan (*SADABS*; Sheldrick, 1996 ▶) *T*_min_ = 0.859, *T*_max_ = 0.942
19255 measured reflections
3138 independent reflections
2653 reflections with *I* > 2σ(*I*)
*R*_int_ = 0.026

#### Refinement

*R*[*F* ^2^ > 2σ(*F* ^2^)] = 0.031
*wR*(*F* ^2^) = 0.077
*S* = 1.04
3138 reflections
198 parameters
H atoms treated by a mixture of independent and constrained refinement
Δρ_max_ = 0.49 e Å^−3^
Δρ_min_ = −0.19 e Å^−3^

### 

Data collection: *SMART* (Bruker, 2003 ▶); cell refinement: *SAINT* (Bruker, 2003 ▶); data reduction: *SAINT*; program(s) used to solve structure: *SHELXS97* (Sheldrick, 2008 ▶); program(s) used to refine structure: *SHELXL97* (Sheldrick, 2008 ▶); molecular graphics: *Mercury* (Macrae *et al.*, 2008 ▶); software used to prepare material for publication: *publCIF* (Westrip, 2010 ▶).

## Supplementary Material

Crystal structure: contains datablock(s) I, global. DOI: 10.1107/S1600536811041377/su2322sup1.cif

Supplementary material file. DOI: 10.1107/S1600536811041377/su2322Isup2.mol

Structure factors: contains datablock(s) I. DOI: 10.1107/S1600536811041377/su2322Isup3.hkl

Supplementary material file. DOI: 10.1107/S1600536811041377/su2322Isup4.cml

Additional supplementary materials:  crystallographic information; 3D view; checkCIF report

## Figures and Tables

**Table 1:** Hydrogen-bond geometry (Å, °)

*D*—H⋯*A*	*D*—H	H⋯*A*	*D*⋯*A*	*D*—H⋯*A*
O11—H11⋯O20	0.84	1.76	2.5947 (18)	173
O14—H14⋯O4^i^	0.84	2.03	2.8381 (18)	161
O20—H20*A*⋯O1^ii^	0.79 (3)	2.12 (3)	2.914 (2)	177 (3)
O20—H20*B*⋯O11^iii^	0.74 (3)	2.06 (3)	2.7899 (19)	169 (3)
C6—H6⋯O14^iv^	0.95	2.48	3.209 (2)	134

Symmetry codes: (i) 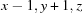; (ii) 
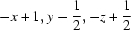
; (iii) 
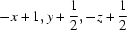
; (iv) 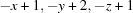.

**Fig. 1.**
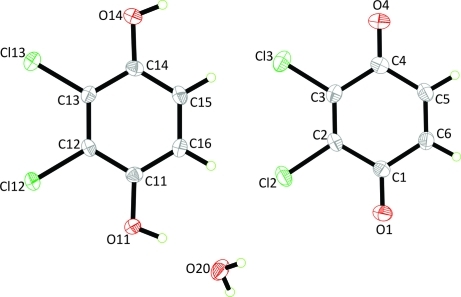
The molecular structure of the title compound, with atom labels and displacement ellipsoids drawn at the 50% probability level [left donor (D); right acceptor (A)].

**Fig. 2.**
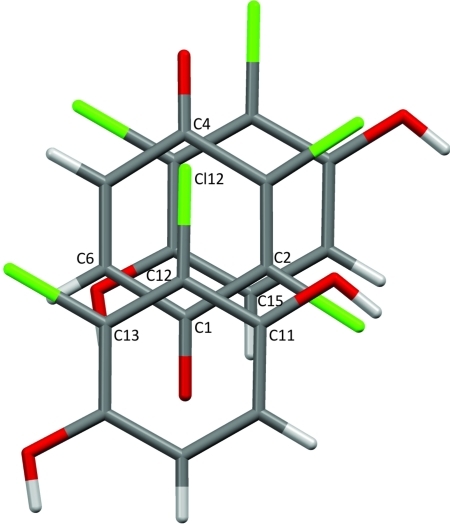
A view perpendicular to the molecular planes, of the stacking of the acceptor (A) and donor (D) molecules in the title compound.

**Fig. 3.**
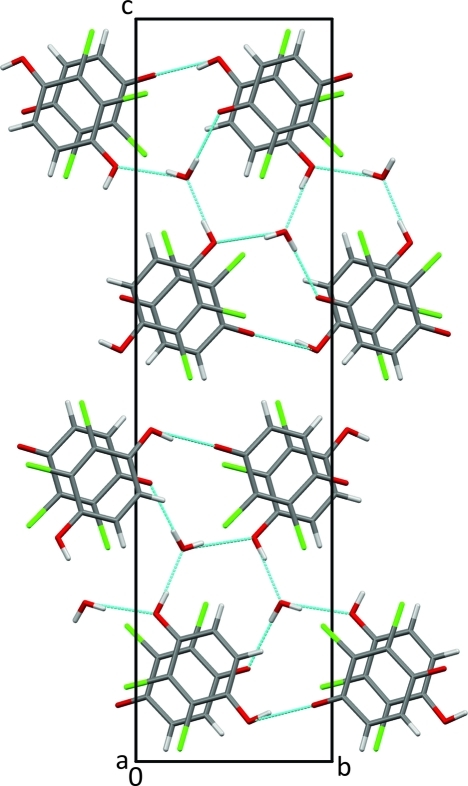
The crystal packing of the title compound, viewed along the a axis. Stacks of donor (D) and acceptor (A) molecules are connected by O-H···O hydrogen bonds (dashed blue lines).

## supplementary crystallographic information

### Comment

Quinones as electron acceptors (A) and their reduced forms, semiquinones and dihydroquinones as electron donors (D) represent prototypical examples of organice redox systems (Yi *et al.*, 2009*a*,*b*). Furthermore, it is certainly true that interactions between aromatic π-systems have long been observed in crystal structures and rationales for the occurrence of π-π interactions have included explanations which are based on electron donor-acceptor [D—A] models. Such charge-transfer (CT) complexes may form when good electron donors and acceptors lie in close proximity, and this situation is typically associated with intense electronic CT transitions in the UV-vis spectral region. Basically, the filled donor molecular orbital (HOMO) and the vacant acceptor molecular orbital (LUMO) maximize their overlap. Quinhydrone, a 1:1 hydroquinone-quinone adduct, is a well known molecular CT complex (Foster, 1969) and its complex stabilization is based on the CT between the electron donor (hydroquinone) and the electron acceptor (quinone). Structural studies of different polymorphs of quinhydrone have been undertaken (Matsuda *et al.*, 1958; Sakurai, 1965, 1968). Quinhydrone analogues have also been studied (Bouvet *et al.*, 2006, 2007, Patil *et al.*, 1984 and Yamamura *et al.*, 2007). A detailed computational study on the stacking energies and the electron density topology in quinhydrone has recently been given (Gonzalez Moa *et al.*, 2007). Additionally, hydrogen bonds contribute further stability, both in the solid state as well as in solution. We report herein on the crystal structure of the title quinhydrone analogue.

The molecular structure of the title compound is illustrated in Fig. 1. The 2,3-Dichloro-1,4-hydroquinone donor (D) and the 2,3-Dichloro-1,4-benzoquinone acceptor (A) molecules form alternate stacks along the [100] direction with their molecular planes [max. deviations for non-H atoms: (D) = 0.0133 (14)Å and (A) = 0.0763 (14)Å ] are tilted by ca. 22.8° and 23.1° about the [100] direction. Their molecular planes are inclined to one another by 1.45 (3)° and are thus almost parallel (Fig. 2). The π–π interactions involving the six-membered rings of the (D) [C11-C16] and (A) [C1-C6] molecules have centroid-centroid distances of 3.5043 (9) Å [D···A^i^; symmetry code (i) x-1, y, z] and 3.9548 (9)Å [D···A].

Intermolecular O—H···O hydrogen bonds between water and the hydroxyl and ketone groups of adjacent stacks form a complex two-dimensional network lying parallel to (001) [Fig. 3]. Chlorine atoms, Cl2 and Cl3^i^, of (A) in adjacent stacks are in short contact with a distance of 3.3902 (7) Å [symmetry code: (i) = 2-x, 0.5+y, 0.5-z]. The two-dimensional networks are linked via C-H···O contacts to form a three-dimensional structure.

### Experimental

Deep-red coloured, elongated (up to 5 mm long) plate-shaped crystals of the 1:1 donor-acceptor adduct [D—A].H_2_O (D = 2,3-Dichloro-1,4-hydroquinone; A = 2,3-Dichloro-1,4-benzoquinone) were formed by slow sublimation of the single component (A) within a closed flask at room-temperature under aerobic conditions.

### Refinement

H atoms of the water molecule were located in a difference Fourier map and refined with *U*_iso_(H) = 1.5 times *U*_eq_(O). Other H atoms were positioned geometrically and refined using a riding model (including free rotation about the hydroxy C—O bond): O—H = 0.84 Å, C—H = 0.95 Å, with *U*_iso_(H) = k × *U*_eq_(O,C), where k = 1.5 for OH H atoms, and k = 1.2 for all other H atoms.

### Crystal data

**Table:**

C_6_H_4_Cl_2_O_2_·C_6_H_2_Cl_2_O_2_·H_2_O	*F*(000) = 752
*M**_r_* = 373.98	*D*_x_ = 1.771 Mg m^−^^3^
Monoclinic, *P*2_1_/*c*	Mo *K*α radiation, λ = 0.71073 Å
Hall symbol: -P 2ybc	Cell parameters from 7958 reflections
*a* = 7.15329 (14) Å	θ = 2.8–27.5°
*b* = 7.19541 (15) Å	µ = 0.86 mm^−^^1^
*c* = 27.2811 (5) Å	*T* = 173 K
β = 92.9738 (18)°	Plate, red
*V* = 1402.29 (5) Å^3^	0.3 × 0.2 × 0.07 mm
*Z* = 4	

### Data collection

**Table:**

Siemens SMART 1K CCD area-detector diffractometer	3138 independent reflections
Radiation source: fine-focus sealed tube	2653 reflections with *I* > 2σ(*I*)
graphite	*R*_int_ = 0.026
rotation method scans	θ_max_ = 27.9°, θ_min_ = 2.9°
Absorption correction: multi-scan (*SADABS*; Sheldrick, 1996)	*h* = −8→9
*T*_min_ = 0.859, *T*_max_ = 0.942	*k* = −9→9
19255 measured reflections	*l* = −35→35

### Refinement

**Table:**

Refinement on *F*^2^	Primary atom site location: structure-invariant direct methods
Least-squares matrix: full	Secondary atom site location: difference Fourier map
*R*[*F*^2^ > 2σ(*F*^2^)] = 0.031	Hydrogen site location: inferred from neighbouring sites
*wR*(*F*^2^) = 0.077	H atoms treated by a mixture of independent and constrained refinement
*S* = 1.04	*w* = 1/[σ^2^(*F*_o_^2^) + (0.0429*P*)^2^ + 0.583*P*] where *P* = (*F*_o_^2^ + 2*F*_c_^2^)/3
3138 reflections	(Δ/σ)_max_ = 0.001
198 parameters	Δρ_max_ = 0.49 e Å^−^^3^
0 restraints	Δρ_min_ = −0.19 e Å^−^^3^

### Special details

**Table:**

Geometry. All s.u.'s (except the s.u. in the dihedral angle between two l.s. planes) are estimated using the full covariance matrix. The cell s.u.'s are taken into account individually in the estimation of s.u.'s in distances, angles and torsion angles; correlations between s.u.'s in cell parameters are only used when they are defined by crystal symmetry. An approximate (isotropic) treatment of cell s.u.'s is used for estimating s.u.'s involving l.s. planes.
Refinement. Refinement of *F*^2^ against ALL reflections. The weighted *R*-factor *wR* and goodness of fit *S* are based on *F*^2^, conventional *R*-factors *R* are based on *F*, with *F* set to zero for negative *F*^2^. The threshold expression of *F*^2^ > σ(*F*^2^) is used only for calculating *R*-factors(gt) *etc*. and is not relevant to the choice of reflections for refinement. *R*-factors based on *F*^2^ are statistically about twice as large as those based on *F*, and *R*- factors based on ALL data will be even larger.

### Fractional atomic coordinates and isotropic or equivalent isotropic displacement parameters (Å^2^)

**Table:**

	*x*	*y*	*z*	*U*_iso_*/*U*_eq_	
C1	0.7866 (2)	0.9165 (2)	0.38598 (7)	0.0220 (4)	
O1	0.72734 (19)	1.07053 (18)	0.37504 (5)	0.0299 (3)	
C2	0.8432 (2)	0.7822 (3)	0.34779 (6)	0.0216 (4)	
Cl2	0.81893 (7)	0.85660 (7)	0.288391 (16)	0.03021 (13)	
C3	0.9107 (2)	0.6140 (3)	0.36016 (6)	0.0220 (4)	
Cl3	0.97542 (7)	0.45526 (7)	0.317735 (18)	0.03252 (13)	
C4	0.9362 (2)	0.5565 (2)	0.41269 (7)	0.0229 (4)	
O4	1.00840 (19)	0.40868 (18)	0.42426 (5)	0.0305 (3)	
C5	0.8731 (2)	0.6879 (3)	0.44963 (7)	0.0252 (4)	
H5	0.8808	0.6525	0.4832	0.030*	
C6	0.8052 (3)	0.8555 (3)	0.43743 (7)	0.0251 (4)	
H6	0.7682	0.9370	0.4625	0.030*	
C11	0.3767 (2)	0.7229 (2)	0.33596 (6)	0.0194 (3)	
O11	0.43186 (18)	0.60154 (17)	0.30131 (4)	0.0245 (3)	
H11	0.4304	0.6550	0.2739	0.037*	
C12	0.3915 (2)	0.6700 (2)	0.38514 (6)	0.0185 (3)	
Cl12	0.47890 (6)	0.45165 (6)	0.399712 (15)	0.02360 (11)	
C13	0.3366 (2)	0.7902 (2)	0.42204 (6)	0.0196 (3)	
Cl13	0.35592 (7)	0.72408 (6)	0.482946 (16)	0.02805 (12)	
C14	0.2652 (2)	0.9655 (2)	0.40960 (6)	0.0206 (4)	
O14	0.2109 (2)	1.07750 (18)	0.44656 (5)	0.0289 (3)	
H14	0.1681	1.1775	0.4347	0.043*	
C15	0.2513 (2)	1.0172 (2)	0.36060 (6)	0.0220 (4)	
H15	0.2035	1.1366	0.3519	0.026*	
C16	0.3058 (2)	0.8979 (2)	0.32418 (6)	0.0213 (4)	
H16	0.2947	0.9361	0.2908	0.026*	
O20	0.4387 (2)	0.7413 (2)	0.21375 (5)	0.0325 (3)	
H20A	0.393 (3)	0.691 (4)	0.1903 (10)	0.049*	
H20B	0.472 (4)	0.834 (4)	0.2061 (10)	0.049*	

### Atomic displacement parameters (Å^2^)

**Table:**

	*U*^11^	*U*^22^	*U*^33^	*U*^12^	*U*^13^	*U*^23^
C1	0.0203 (8)	0.0230 (9)	0.0224 (9)	−0.0010 (7)	−0.0011 (7)	0.0017 (7)
O1	0.0371 (7)	0.0239 (7)	0.0283 (7)	0.0054 (6)	−0.0032 (6)	0.0030 (5)
C2	0.0203 (8)	0.0282 (9)	0.0160 (8)	−0.0027 (7)	−0.0002 (7)	0.0021 (7)
Cl2	0.0329 (2)	0.0399 (3)	0.0178 (2)	0.0027 (2)	0.00070 (17)	0.00536 (19)
C3	0.0190 (8)	0.0246 (9)	0.0226 (9)	−0.0017 (7)	0.0023 (7)	−0.0043 (7)
Cl3	0.0360 (3)	0.0321 (3)	0.0298 (3)	0.0031 (2)	0.00496 (19)	−0.00926 (19)
C4	0.0195 (8)	0.0235 (9)	0.0254 (9)	−0.0020 (7)	−0.0005 (7)	0.0027 (7)
O4	0.0315 (7)	0.0243 (7)	0.0356 (8)	0.0055 (6)	−0.0008 (6)	0.0056 (6)
C5	0.0266 (9)	0.0297 (9)	0.0193 (9)	0.0005 (8)	0.0012 (7)	0.0028 (7)
C6	0.0267 (9)	0.0281 (9)	0.0206 (9)	0.0021 (8)	0.0022 (7)	−0.0022 (7)
C11	0.0206 (8)	0.0185 (8)	0.0190 (8)	−0.0021 (7)	0.0013 (6)	−0.0019 (6)
O11	0.0373 (7)	0.0196 (6)	0.0169 (6)	0.0037 (5)	0.0047 (5)	0.0002 (5)
C12	0.0188 (8)	0.0144 (7)	0.0223 (9)	0.0003 (6)	−0.0001 (6)	0.0023 (6)
Cl12	0.0305 (2)	0.0166 (2)	0.0236 (2)	0.00314 (17)	0.00040 (17)	0.00279 (16)
C13	0.0214 (8)	0.0210 (8)	0.0163 (8)	−0.0018 (7)	0.0002 (6)	0.0022 (7)
Cl13	0.0374 (3)	0.0288 (2)	0.0179 (2)	0.0034 (2)	0.00093 (17)	0.00261 (17)
C14	0.0219 (8)	0.0178 (8)	0.0221 (9)	−0.0007 (7)	0.0021 (7)	−0.0025 (7)
O14	0.0412 (8)	0.0213 (6)	0.0245 (7)	0.0081 (6)	0.0043 (6)	−0.0023 (5)
C15	0.0241 (9)	0.0161 (8)	0.0257 (9)	0.0005 (7)	−0.0009 (7)	0.0034 (7)
C16	0.0232 (9)	0.0220 (8)	0.0186 (9)	−0.0001 (7)	0.0004 (7)	0.0041 (7)
O20	0.0539 (9)	0.0238 (7)	0.0192 (7)	−0.0103 (7)	−0.0027 (6)	0.0009 (5)

### Geometric parameters (Å, °)

**Table:**

C1—O1	1.218 (2)	C11—C12	1.393 (2)
C1—C6	1.470 (3)	O11—H11	0.8400
C1—C2	1.493 (2)	C12—C13	1.400 (2)
C2—C3	1.340 (3)	C12—Cl12	1.7292 (16)
C2—Cl2	1.7070 (17)	C13—C14	1.396 (2)
C3—C4	1.494 (2)	C13—Cl13	1.7270 (17)
C3—Cl3	1.7069 (18)	C14—O14	1.363 (2)
C4—O4	1.217 (2)	C14—C15	1.386 (2)
C4—C5	1.470 (3)	O14—H14	0.8400
C5—C6	1.335 (3)	C15—C16	1.384 (2)
C5—H5	0.9500	C15—H15	0.9500
C6—H6	0.9500	C16—H16	0.9500
C11—O11	1.360 (2)	O20—H20A	0.79 (3)
C11—C16	1.389 (2)	O20—H20B	0.74 (3)
			
O1—C1—C6	121.26 (17)	C16—C11—C12	118.64 (16)
O1—C1—C2	121.44 (16)	C11—O11—H11	109.5
C6—C1—C2	117.30 (15)	C11—C12—C13	120.89 (15)
C3—C2—C1	121.09 (15)	C11—C12—Cl12	118.58 (13)
C3—C2—Cl2	122.70 (14)	C13—C12—Cl12	120.53 (13)
C1—C2—Cl2	116.21 (13)	C14—C13—C12	119.78 (15)
C2—C3—C4	121.08 (16)	C14—C13—Cl13	119.55 (13)
C2—C3—Cl3	122.71 (14)	C12—C13—Cl13	120.68 (13)
C4—C3—Cl3	116.20 (13)	O14—C14—C15	123.05 (15)
O4—C4—C5	121.72 (17)	O14—C14—C13	117.98 (15)
O4—C4—C3	121.32 (17)	C15—C14—C13	118.96 (16)
C5—C4—C3	116.95 (15)	C14—O14—H14	109.5
C6—C5—C4	122.04 (16)	C16—C15—C14	121.10 (16)
C6—C5—H5	119.0	C16—C15—H15	119.4
C4—C5—H5	119.0	C14—C15—H15	119.4
C5—C6—C1	121.43 (17)	C15—C16—C11	120.63 (16)
C5—C6—H6	119.3	C15—C16—H16	119.7
C1—C6—H6	119.3	C11—C16—H16	119.7
O11—C11—C16	122.47 (15)	H20A—O20—H20B	108 (3)
O11—C11—C12	118.89 (15)		
			
O1—C1—C2—C3	179.07 (17)	O11—C11—C12—C13	179.97 (15)
C6—C1—C2—C3	−0.9 (2)	C16—C11—C12—C13	−0.1 (3)
O1—C1—C2—Cl2	−0.3 (2)	O11—C11—C12—Cl12	−0.2 (2)
C6—C1—C2—Cl2	179.69 (13)	C16—C11—C12—Cl12	179.74 (13)
C1—C2—C3—C4	−1.5 (3)	C11—C12—C13—C14	0.2 (3)
Cl2—C2—C3—C4	177.83 (13)	Cl12—C12—C13—C14	−179.57 (13)
C1—C2—C3—Cl3	179.46 (13)	C11—C12—C13—Cl13	−179.82 (13)
Cl2—C2—C3—Cl3	−1.2 (2)	Cl12—C12—C13—Cl13	0.4 (2)
C2—C3—C4—O4	−175.28 (17)	C12—C13—C14—O14	179.13 (15)
Cl3—C3—C4—O4	3.8 (2)	Cl13—C13—C14—O14	−0.8 (2)
C2—C3—C4—C5	3.7 (2)	C12—C13—C14—C15	−0.4 (3)
Cl3—C3—C4—C5	−177.23 (13)	Cl13—C13—C14—C15	179.68 (13)
O4—C4—C5—C6	175.42 (18)	O14—C14—C15—C16	−179.12 (16)
C3—C4—C5—C6	−3.5 (3)	C13—C14—C15—C16	0.4 (3)
C4—C5—C6—C1	1.2 (3)	C14—C15—C16—C11	−0.2 (3)
O1—C1—C6—C5	−178.88 (17)	O11—C11—C16—C15	180.00 (16)
C2—C1—C6—C5	1.1 (3)	C12—C11—C16—C15	0.0 (3)

### Hydrogen-bond geometry (Å, °)

**Table:**

*D*—H···*A*	*D*—H	H···*A*	*D*···*A*	*D*—H···*A*
O11—H11···O20	0.84	1.76	2.5947 (18)	173
O14—H14···O4^i^	0.84	2.03	2.8381 (18)	161
O20—H20A···O1^ii^	0.79 (3)	2.12 (3)	2.914 (2)	177 (3)
O20—H20B···O11^iii^	0.74 (3)	2.06 (3)	2.7899 (19)	169 (3)
C6—H6···O14^iv^	0.95	2.48	3.209 (2)	134

Symmetry codes: (i) *x*−1, *y*+1, *z*; (ii) −*x*+1, *y*−1/2, −*z*+1/2; (iii) −*x*+1, *y*+1/2, −*z*+1/2; (iv) −*x*+1, −*y*+2, −*z*+1.
